# Evaluation of efficacy of transcatheter arterial chemoembolization combined with computed tomography-guided radiofrequency ablation for hepatocellular carcinoma using magnetic resonance diffusion weighted imaging and computed tomography perfusion imaging: A prospective study

**DOI:** 10.1097/MD.0000000000005518

**Published:** 2017-01-20

**Authors:** Guo-Liang Shao, Jia-Ping Zheng, Li-Wen Guo, Yu-Tang Chen, Hui Zeng, Zheng Yao

**Affiliations:** Department of Radiology, Zhejiang Cancer Hospital, Hangzhou, P. R. China.

**Keywords:** computed tomography perfusion imaging, computed tomography-radiofrequency ablation, efficacy, hepatocellular carcinoma, magnetic resonance diffusion weighted imaging, prognosis, transcatheter arterial chemoembolization

## Abstract

**Background:**

The purpose of this study is to evaluate the efficacy of transcatheter arterial chemoembolization (TACE) combined with computed tomography-guided radiofrequency ablation (CT-RFA) in the treatment of hepatocellular carcinoma (HCC) using magnetic resonance diffusion weighted imaging (MR-DWI) and CT perfusion imaging (CT-PI).

**Methods:**

From January 2008 to January 2014, a total of 522 HCC patients receiving TACE combined with CT-RFA were included in this study. All patients underwent TACE followed by CT-RFA, and 1 day before treatment and 1 month after treatment they received MR-DWI and CT-PI. Enzyme-linked immunosorbent assay (ELISA) was performed to detect the concentration of alpha-fetoprotein (AFP). Tumor response was evaluated using the revised RECIST criteria. One-year follow-up was conducted on all patients. Receiver-operating characteristic (ROC) curve was drawn to evaluate the efficacy of TACE combined with CT-RFA for HCC using MR-DWI and CT-PI.

**Results:**

Total effective rate (complete remission [CR] + partial remission [PR]) of TACE combined with CT-RFA for HCC was 82.95%. HCC patients of CR + PR had lower hepatic blood flow (HBF), hepatic blood volume (HBV), permeability surface (PS), hepatic arterial perfusion (HAP), and hepatic perfusion index (HPI) levels than those of SD + PD, but HCC patients of CR + PR had higher mean transit time (MTT) level than those of SD + PD. The patients of PR + CR had higher apparent diffusion coefficient (ADC) values than those of SD + PD. The patients of PR + CR showed lower AFP concentration than those of SD + PD. ROC curve analysis indicated that the area under the curve (AUC) of AFP, HBV, PS, HAP, HPI, and ADC was more than 0.7, but the AUC of HBF, MTT, and PVP were less than 0.7. After treatment, the AFP, HBF, HBV, PS, HAP, and HPI in the HCC patients with recurrence were higher than those in the HCC patients without, but MTT and ADC in the HCC patients with recurrence were lower than those in the HCC patients without.

**Conclusion:**

These findings indicate that MR-DWI and CT-PI can effectively evaluate the efficacy of TACE combined with CT-RFA and postoperative recurrence of HCC.

## 1 Introduction

Primary liver cancer consists of 2 subtypes, hepatocellular carcinoma (HCC) and intrahepatic cholangiocarcinoma.^[1]^ As HCC accounts for 85% to 90% of all primary liver cancer, the 2 entities are often interchangeably used.^[2]^ HCC is the 3rd most common cause of cancer mortality worldwide.^[3]^ Over 75% cases of HCC occur in the Asia-Pacific region, of which more than a half occur in China alone.^[4]^ Because of asymptomatic features, most HCC patients are diagnosed at late stage, and featured by large tumor size and unresectable lesions.^[5]^ Potential curative treatments for HCC included ablation, resection, and liver transplantation, which however have been performed for fewer than 20% HCC patients, mainly for complicating cirrhosis and/or advanced stage of cancer at diagnosis.^[6]^ Therefore, it is of great significance to evaluate the efficacy and prognostic value of new treatments for HCC. Radiofrequency ablation (RFA) and transcatheter arterial chemoembolization (TACE) are minimally invasive options applied individually or in combination to achieve the balance in successful tumor eradication and maximal preservation of liver function.^[7]^ RFA is considered as the effective treatment of choice only for patients with early-stage small HCC (≤2.0 cm).^[8–10]^ Therefore, RFA combined with TACE is increasingly advocated to improve local tumor control for larger nodules.^[11]^

Magnetic resonance imaging is a sensitive technique for necrosis detection for its superior tissue contrast and the nonviable tissue appears as high signal areas on the T2 weighted images.^[12]^ Magnetic resonance diffusion weighted imaging (MR-DWI) is highly sensitive to the random motion of water protons and reflects their interactions with cell membranes and macromolecules.^[13]^ The apparent diffusion of water in tissues can reflect tissue cellularity, tortuosity of extracellular space, viscosity of fluids, and integrity of cell membranes.^[14]^ Apparent diffusion coefficient (ADC) is a quantitative measure of tissue diffusivity, making it possible to identify objectively treatment response by comparing results.^[13]^ MR-DWI has been applied to multiple solid tumors, including pancreatic cancer peripheral zone prostate cancer, locally advanced rectal cancer, for the detection of changes in cellularity, which is indirectly measured by an increase in the ADC of water molecules within lesions.^[15–18]^ Conventional computed tomography (CT) has become the main diagnostic tool in tumor evaluation, including diagnosis, staging, or monitoring of anticancer therapies and CT perfusion imaging (CT-PI) clinically augments the standard therapeutic assessment.^[19]^ CT-PI, using standard nonionic iodinated contrast, is a bolus-tracking technique performed on multi-detector (-helical) CT scanners popularly servicing emergency departments.^[20,21]^ CT-PI has lower signal to noise compared with magnetic resonance imaging, which necessitates critical postprocessing (smoothing) of the data.^[22]^ Many studies have demonstrated correlation between tumor perfusion parameters and invasive histologic biomarkers of angiogenesis and the application of CT-PI in detection of cancers, including abdominal cancer, pancreatic endocrine tumors, and advanced nonsmall cell lung cancer.^[19,23–25]^ In the present study, we tried to evaluate the efficacy of TACE combined with computed tomography-guided radiofrequency ablation (CT-RFA) in the treatment of HCC using MR-DWI and CT-PI.

## 2 Materials and methods

### 2.1 Study subjects

A total of 522 patients with primary HCC were recruited for the present study, who were admitted at Zhejiang Cancer Hospital from January 2008 to January 2014, including 328 males and 192 females, with mean age of 53.80 ± 9.50 years old, ranging from 26 to 79 years old. Inclusion criteria: confirmed as primary HCC by clinical manifestation and histopathological examination on liver biopsy specimens; with poor liver function or tumor greater than 5 cm in the maximum diameter; and not yet received chemoradiotherapy therapy. Exclusion criteria: with severe dysfunction of heart, liver or kidney; with combined immune and endocrine system diseases; and with history of malignant tumor in other organs and liver metastasis. All the patients underwent TACE, after which CT-RFA was performed immediately. MR-DWI and CT-PI were conducted 1 day before and 1 month after the treatment. Enzyme-linked immunosorbent assay (ELISA) was selected to detect the concentration of alpha-fetoprotein (AFP). The normal liver tissues in nontumor area of all patients were taken as the normal control. This study has been approved by the Ethics Committee of Zhejiang Cancer Hospital, and all the patients have been informed and signed informed consent.

### 2.2 Transcatheter hepatic arterial chemoembolization (TACE)

One day before TACE treatment, all the patients underwent routine examination, including liver and renal function, blood, urine, and stool examination, and electrocardiograph. TACE therapeutic regimen: after local anesthesia, the Seldinger technique was applied for routine puncture in femoral artery; when the catheter reached the feeding arteries of the tumors, 5-fluorouracil (750–1000 mg), mitomycin (20 mg), and cisplatin (60 mg) were infused. Then cisplatin (20 mg) combined with 48% super liquefied lipiodol (10–20 mL) were added to embolize the feeding arteries of the tumors; in patients with large lesions, gelatin sponge was used to strengthen embolization until the arteries of tumors stopped feeding. The patients received postoperative liver protection therapy with reduced glutathione.

### 2.3 CT-guided radiofrequency ablation (CT-RFA)

The 128-slice spiral CT scanner was from the General Electric Company (Schenectady, NY). The scanning parameters were 120 kV, 200 to 300 mA, 5 mm slice thickness, and 1.375 screw pitch. The bipolar CT-RFA system was from Olympus Optical Co., Ltd. (Tokyo, Japan); the ablation probe was 1.8 mm in diameter, and the effective ablation area of a single electrode was 45 mm × 35 mm. Before CT-RFA, an enhanced CT scanning was performed to detect the tumor location. On the basis of lesion size and lipiodol deposition, appropriate puncture point and approach and ablation plan were decided. According to the tumor location, the patients were fixed supine, lateral, or prostrate. After local anesthesia, the RF probe was inserted into the predetermined target under the CT guidance. Ablation was performed in accordance with the CT-RFA procedures. When necessary, timely adjustment was made of CT-RFA parameters and the position and depth of the RF probe. The ablation was performed step by step from where was far to the epidermis to where was near. On the premise of no damage to the adjacent organs, the ablation was supposed to cover a range of lesions as large as possible. At the end of the ablation, the needle was withdrawn and needle tract bleeding was stemmed conventionally. After the treatment, the patients were given an electrocardiograph monitoring for 4 hours and routine antiinflammatory drugs.

### 2.4 CT perfusion imaging (CT-PI)

The AS 128-slice spiral mode CT scanner (Siemens Company, Erlangen, Germany) was applied for CT-PI. The scanning parameters were 120 kV, 30 mA, 5 mm slice thickness, 0 layer spacing, and 2.5 mm collimation. The scanning mode: 4 layers were scanned at the same time with 1 rotation of bulb tube (1 second) and the scanned area covered the whole liver. After a conventional CT scanning, an appropriate perfusion level was selected. Then, patients were given an intravenous injection of 50 mL of nonionic contrast agent iopromide 370 (ultravist 370 mgI/mL, Guangzhou Schering Pharmaceutical Co., Ltd., Guangzhou, China) at 4 mL/s. Eight seconds after contrast injection, the scanning was performed for 50 seconds, collecting 200 images.

### 2.5 Magnetic resonance diffusion weighted imaging (MR-DWI)

The 1.5 T superconducting magnetic resonance scanner (General Electric Company) was applied for MR-DWI. The scanning parameters were TR/TE = 8000/75 ms, 6 mm slice thickness, 1.5 mm interval, 40 × 40 mm scanning field, and 128 × 128 matrix. The scanning was performed with 4 different diffusion sensitivity coefficients (b-value; b = 0, 500, 800, 1000 s/mm^2^, respectively) in X, Y, Z axes. Echo planar imaging was applied as scanning sequence. Frequency-selective fat-saturation pulse (Fat-Sat) technique was conventionally performed. The scanning range was the same as that of conventional T2, and eupnea of patients were required during the scanning. The scanning lasted for 5 minutes with 6 times of data acquisition and signal-to-noise ratio of 1.0.

### 2.6 Imaging analysis

CT-PI analysis: the results of CT-PI were postprocessed by Siemens Nixdorf computer programmed in MED-PC (Med Associates, Fairfield, VT) on the workstation. The liver perfusion mode was applied, with the threshold range of −50 to 150 Hu, abdominal aorta as the input artery, trunk or its branch of portal vein as the input vein, and default value for the left. The computer then automatically generated the time-density curve, and calculated the hepatic blood flow (HBF), hepatic blood volume (HBV), mean transit time (MTT), permeability surface (PS), hepatic arterial perfusion (HAP), portal vein perfusion (PVP), and hepatic perfusion index (HPI). Corresponding perfusion parameters of the tumor tissue and normal liver tissue were measured using region of interest (ROI) in the perfusion map. The range of ROI selection should be as large as possible for the active tumor lesions, where lipiodol deposition was not much and accompanied with obviously enhanced local tissue. Large vessels and necrotic tissues were avoided. For normal liver tissue, the region 2 to 5 cm away from the tumor margin was selected.

MR-DWI analysis: the MR-DWI was analyzed by the workstation of the 1.5 T magnetic resonance scanner (General Electric Company). On each surface of the lesion, an ROI was selected which was slightly smaller than the tumor range. The ADC was measured from each ROI and the mean ADC was taken as the ADC of the lesion; the ADC of the normal liver tissue was obtained for control.

### 2.7 Enzyme-linked immunosorbent assay (ELISA)

Venous blood (5 mL, empty stomach) was extracted in the morning and stored in separation tube. Then, serum was separated using refrigerated centrifuge at 4 °C and stored at −20 °C in liquid nitrogen for preparation. The AFP ELISA kit was purchased from the Zhengzhou Auto-Biotechnology Ltd. (Henan, China), and the ELISA reader was bought from the Rayto Life and Analytical Sciences Co., Ltd. (Shenzhen, China). The AFP was detected in accordance with the operating instruction of kit. The critical value of AFP was 20 ng/mL.

### 2.8 Efficacy evaluation

Efficacy was evaluated according to the standard of solid tumor ablation^[26]^ and based on the results of the enhanced CT scanning. Patients of the 2 groups received enhanced CT scanning before TACE and 1 month after CT-RFA, respectively; on the basis of whether the lesions were strengthened (the tumor necrosis area was the area not reinforced), the necrosis area and volume of the tumor were calculated. According to the proportion of the necrosis area and volume of the tumor, the efficacy was divided into: complete remission (CR) – the tumor tissues were completely necrotic; partial remission (PR) – the tumor necrosis ratio was more than 80%; stabilization of disease (SD) – the tumor necrosis ratio was more than 50%; and progressive disease (PD) – the tumor was enlarged or new tumor emerged. CR and PR were regarded effective and SD and PD ineffective.

### 2.9 Follow-up

A 1-year follow-up was carried out for all the patients. According to tumor recurrence situation, the patients were grouped into the recurrence group and the nonrecurrence group. The follow-up started from the time when the patients were clearly diagnosed to January 31, 2015. The follow-up was conducted every 2 months and mainly by review or telephone. No lost cases. Follow-up methods included MR-DWI, CT-PI, and enhanced CT scanning.

### 2.10 Statistical analysis

Measurement data were represented as x ± s. Comparison of measurement data were performed using *t* test. Count data were presented as ratio or percentage and χ^2^ test was performed to compare the count data. Receiver-operating characteristic (ROC) curve was applied to analyze the evaluation value of MR-DWI and CT-PI for the efficacy of TACE and CT-RFA on the treatment of HCC. *P* < 0.05 was considered statistically significant.

## 3 Results

### 3.1 Efficacy of TACE combined with CT-RFA for HCC patients

The TACE and CT-RFA were successfully performed in all the 522 patients. Efficiency evaluation results showed there were 135 cases of CR patients, 298 PR patients, 56 SD patients, and 33 PD patients. The total efficiency of TACE combined with CT-RFA reached 82.95%.

### 3.2 Comparison of CT-PI parameter of the HCC patients before and after TACE combined with CT-RFA

**Figure 1:**
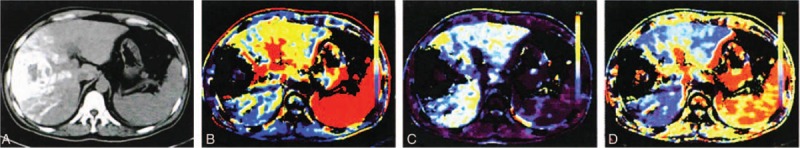
Lipiodol deposition after CT-RFA. (A) Uniformity of lipiodol deposition using CT plain scanning; (B) high blood perfusion in lipiodol sparse region by HAP map; (C) no blood perfusion in lipiodol concentrated region by PVP map; and (D) blood perfusion in lipiodol sparse region by HPI map. CT = computed tomography, CT-RFA = CT-guided radiofrequency ablation, HAP = hepatic arterial perfusion, HPI = hepatic perfusion index, PVP = portal vein perfusion.

Observed from the manifestations of CT-PI which was performed after the CT-RFA, lipiodol was densely filled in 177 cases, unevenly filled in 345 cases; no blood perfusion was found in region with high concentration of lipiodol, while relatively high hepatic arterial infusion was found where lipiodol was sparse (Fig. 1). Comparing the CT-PI parameters of target lesions before and after the treatment, we found that HBF, HBV, PS, HAP, and HPI in patients before treatment were significantly higher than those in the normal liver tissue (all *P* < 0.05); compared to the levels before treatment, these elements significantly decreased after treatment and were still significantly higher than the normal liver tissue (all *P* < 0.05). But MTT was significantly lower than that in the normal liver tissue both before and after treatment, though it significantly increased after treatment as compared to the level before treatment (all *P* < 0.05). PVP showed no significant difference (both *P* > 0.05) (Table 1). Comparing the CT-PI parameters of target lesions in patients with different efficacy after the CT-RFA, we found that the patients of CR + PR showed significantly lower HBF, HBV, PS, HAP, and HPI than those of SD + PD (all *P* < 0.05), indicating that HBF, HBV, PS, HAP, and HPI were negatively related with the postoperative effects; but significantly higher MTT was observed in the patients of CR + PR than those of SD + PD (*P* < 0.05), indicating that MTT was positively related with the postoperative effect (Table 2).

**Table 1:**
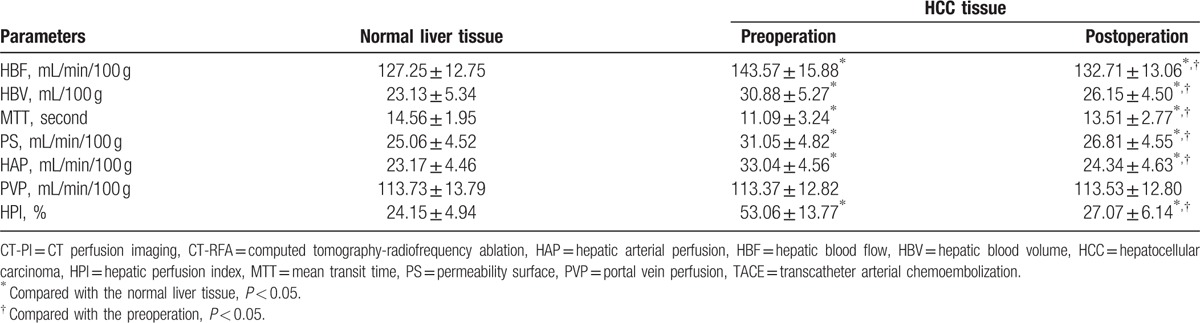
Comparison of CT-PI parameters before and after TACE combined with CT-RFA in HCC patients.

**Table 2:**
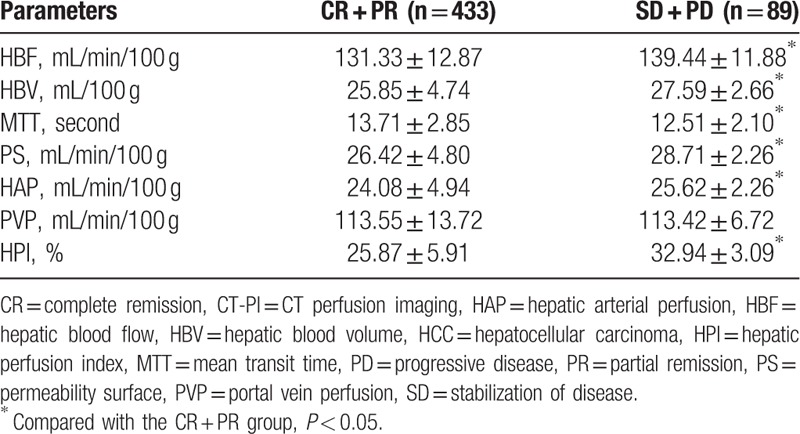
Comparisons of CT-PI parameters in the HCC patients with different efficacy.

### 3.3 Comparison of ADC value and MR-DWI images of the HCC patients with different efficacy before and after TACE combined with CT-RFA

**Figure 2:**
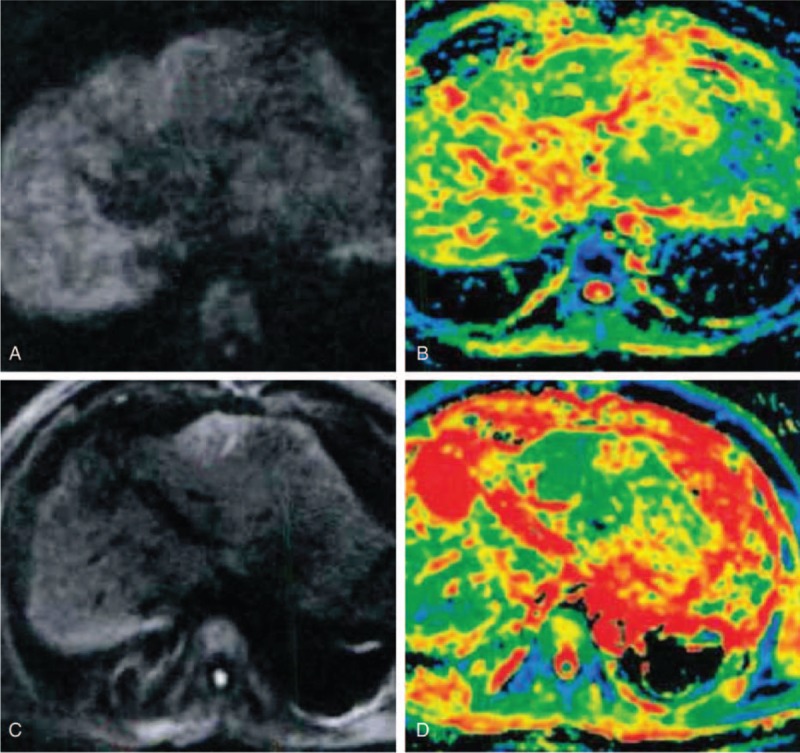
MR-DWI images of HCC patients before and after TACE combined with CT-guided RFA treatment. (A) Homogeneous high MR-DWI signal before 1 day of TACE; (B) significant low signal by ADC before 1 day of TACE; (C) significant decreased MR-DWI signal of the lesion after 1 month of RFA; and (D) significant increased ADC signal of the lesion after 1 month of RFA. ADC = apparent diffusion coefficient, CT = computed tomography, MR-DWI = diffusion weighted magnetic resonance imaging, HCC = hepatocellular carcinoma, RFA = radiofrequency ablation, TACE = transcatheter arterial chemoembolization.

The results of MR-DWI which was performed after the CT-RFA showed that the lesions presented homogeneous high signal in MR-DWI and obvious low signal by ADC, and after the treatment significantly weakened and increased, respectively (Fig. 2). Comparing the ADC values of target lesions before and after the treatment, the ADC value of MR-DWI was significantly lower in target lesions than normal liver tissue both before and after treatment (all *P* < 0.05) although it significantly increased after treatment (all *P* < 0.05) (Table 3). Comparing the ADC values of target lesions before and after the treatment in patients with different efficacy, we found that patients of PR + CR group showed significantly higher ADC value than those of SD + PD group, indicating that ADC value was positively related with the postoperative effects (Table 4).

**Table 3:**
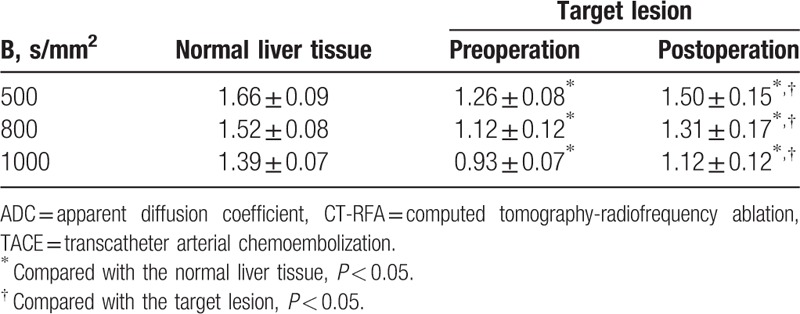
Comparison of ADC values of target lesions and normal liver tissue before and after TACE combined with CT-RFA (×10^–3^ mm^2^/s).

**Table 4:**
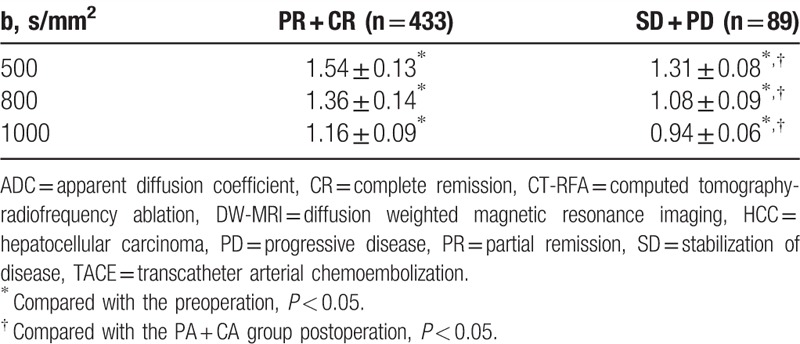
Comparisons of ADC values of DW-MRI in the HCC patients with different efficacy of TACE combined with CT-RFA (×10^–3^ mm^2^/s).

### 3.4 Comparison of AFP concentrations in the HCC patients with different efficacy before and after TACE combined with CT-RFA

**Figure 3:**
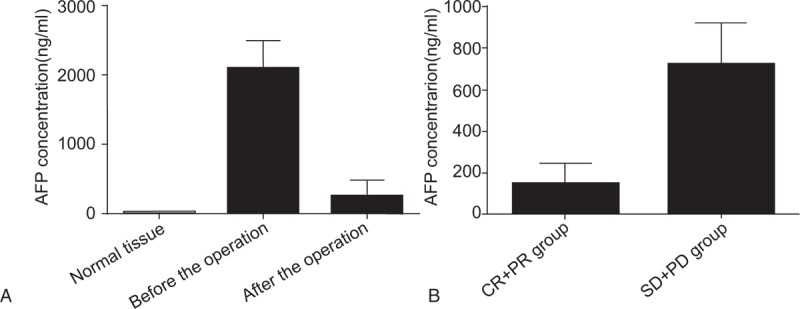
Comparison of AFP concentration before and after TACE combine with CT-guided RFA in patients with different efficacy. (A) Comparison of AFP concentration between the normal liver tissue and target lesion before and after treatment; (B) comparison of AFP concentration among patients with different efficacy. AFP = alpha-fetoprotein, PD = progressive of disease, PR = partial remission, SD = stabilization of disease, TACE = transcatheter arterial chemoembolization.

Comparing the AFP concentrations of target lesion in HCC patients before and after treatment, we found that the AFP concentrations of target lesion were significantly higher than that of the normal liver tissue both before and after treatment (both *P* < 0.05), although it significantly decreased after treatment as compared to the that before treatment (*P* < 0.05) (Fig. 3A). Comparing the AFP concentrations of target lesion in HCC patients, we found that patients of PR + CR showed significantly lower AFP concentration than those of SD + PD, indicating that AFP concentration was negatively related with the postoperative effects (Fig. 3B).

### 3.5 Evaluation of CT-PI parameters, ADC value, and AFP concentration on the efficacy of TACE combined with CT-RFA for the HCC patients

**Figure 4:**
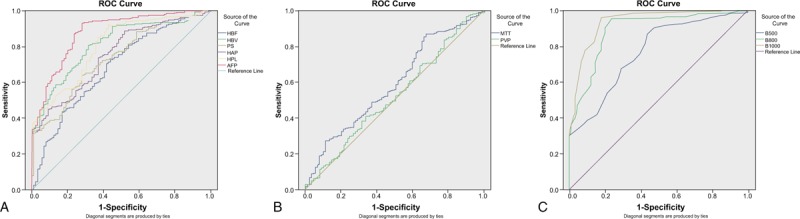
ROC curve analysis of CT-PI parameters and ADC value for evaluating the efficacy of TACE combine with CT-guided RFA for HCC. (A) HBF, HBV, PS, HAP, and HPI; (B) MTT, PVP, and AFP; and (C) ADC value of MR-DWI. ADC = apparent diffusion coefficient, CT-PI = CT perfusion imaging, HAP = hepatic arterial perfusion, HBF = hepatic blood flow, HBV = hepatic blood volume, HCC = hepatocellular carcinoma, HPI = hepatic perfusion index, MR-DWI = diffusion weighted magnetic resonance imaging, MTT = mean transit time, PS = permeability surface, PVP = portal vein perfusion, RFA = radiofrequency ablation, ROC = receiver-operating characteristics.

The ROC curve analyzed the efficacy evaluation of CT-PI parameters, ADC value, and AFP concentration in patients with different efficacy (Fig. 4 and Table 5). It was showed that the area under the ROC curve of the parameters HBV, PS, HAP, HPI, ADC value (500, 800, 1000 s/mm^2^), and AFP were more than 0.7, while that of the parameters HBF, MTT, and PVP was less than 0.7, suggesting that the parameters AFP, HBV, PS, HAP, HPI, and ADC value had medium diagnostic value on the efficacy and the parameters HBF, MTT, and PVP had relative low diagnostic value. The best cut-off value, sensitivity, and specificity of each parameter for diagnosing efficacy were selected using maximum Youden index (Table 5).

**Table 5:**
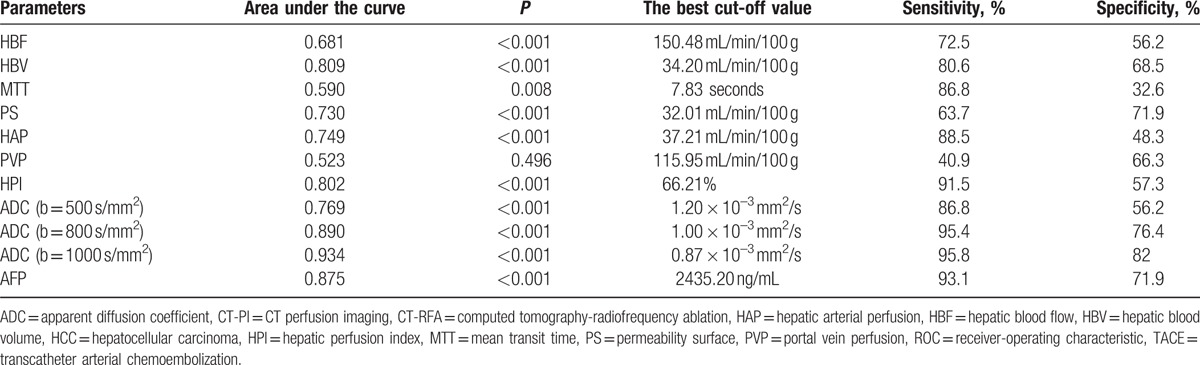
The ROC curve of the ADC values and parameters of CT-PI in the HCC patients with different efficacy of TACE combined with CT-RFA.

### 3.6 Correlations of CT-PI parameters, ADC value, and AFP concentration with the postoperative recurrence of the HCC patients

The 1-year follow-up showed that all the patients of CR had no recurrence, 84 cases had recurrent lesions, among which 34 cases led to final death and 5 cases had new lesions in the liver beyond the treatment areas. Comparing the CT-PI parameters, AFP concentration, and the ADC value of target lesions after the treatment in the patients with or without recurrence, we found that the parameters HBF, AFP, HBV, PS, HAP, and HPI were significantly high in the recurrent patients compared with the nonrecurrent ones, while MTT along with the ADC value significantly decreased (all *P* < 0.05) (Table 6).

**Table 6:**
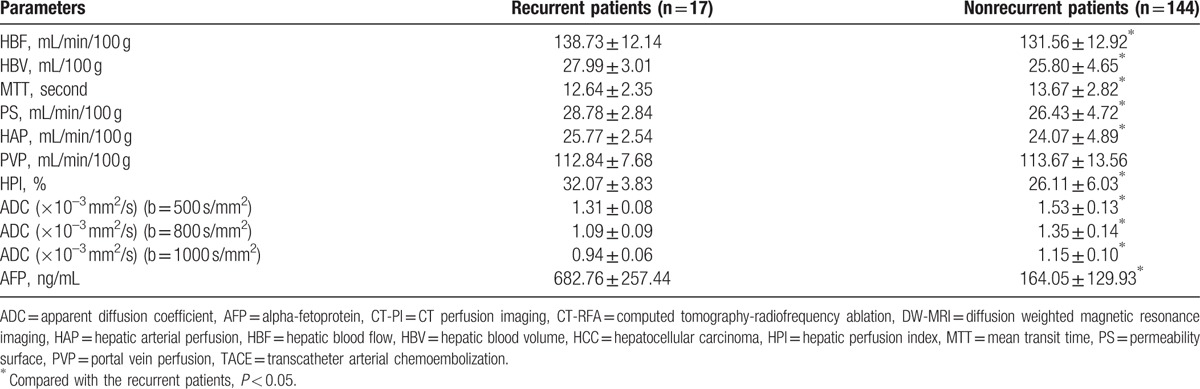
Comparisons of parameters of CT-PI and ADC values of DW-MRI of the HCC patients after TACE combined with CT-RFA in the recurrent and nonrecurrent patients (×10^–3^ mm^2^/s).

## 4 Discussion

In the study, the roles of MR-DWI and CT-PI were investigated in HCC patients for the evaluation of the efficacy and prognostic value of TACE combined with CT-RFA. In view of the above results, we found that 433 cases of all the 522 patients were determined with CR or PR, indicating the high efficiency of the treatment. Local tumor control and survival are important parameters used to evaluate the efficacy of the treatments for HCC.^[10]^ Cho et al^[27]^ reported that CT-RFA showed an improved 3-year survival status for patients with HCC. Shiina et al^[28]^ demonstrated that CT-RFA was locally curative for HCC and led to a survival as long as 10 years for early-stage HCC. Veltri et al^[29]^ recommended a therapy combined CT-RFA and TACE for unresectable nonearly HCC. Besides, the ADC value on MR-DWI and parameters of CT-PI were the target of our study to assess their value in evaluating the efficacy and prognosis of TACE combined with CT-RFA for HCC.

In the present study, we found that compared with before the TACE combined with CT-RFA treatment, the parameters of CT-PI, HBF, HBV, PS, HAP, and HPI decreased and MTT increased significantly; the ADC values of target lesions increased obviously. The changes of the above-mentioned indices hinted their indicative function. Specifically, according to the results, the better the efficacy, the lower the HBV, PS, HAP, and HPI, and the higher the MTT, and the higher the ADC value with the same b-value. The ROC curve therefore identified that HBV, PS, HAP, AFP, and HPI had medium value in assessing the efficacy of the treatment, as well as the ADC value on MR-DWI when the b-value was set at 500 or 800 s/mm^2^. Moreover, we have found that AFP concentration significantly decreased after treatment and patients with PR + CR showed higher AFP concentration than those with SD + PD, suggesting that AFP was negatively related with the postoperative effect. As for prognosis analysis, we also found that HBV, PS, HAP, and HPI of target lesions were relatively higher while MTT and ADC value lower in recurrent patients than in nonrecurrent patients after the treatment, suggesting their indicating value.

Changes in perfusion parameters are recognized of great value in qualitative and differential diagnosis of HCC and CT-PI can detect the abnormality of liver perfusion before morphologic change occurs.^[30]^ As changes at imaging link to those in tumor pathophysiology, parameters of CT-PI can represent the alteration of blood flow (BF), blood volume (BV) and permeability in tumor vessels secondary to their structural and functional abnormalities.^[19]^ It was reported that the HBV, PS, HAP and HPI were found higher in HCC tissue compared with in background liver tissue, but MTT was decreased.^[30–32]^ Kan et al. reported that MTT was increased after TACE because of obstruction of tumor vessels and reduced BF after TACE.^[33]^ Hypoxia induced by TACE stimulates tumor angiogenesis and increases vascular permeability, suggesting that tumor permeability after TACE was of significance for assessing the efficacy.^[30]^ Therefore, the decreased HBV, PS, HAP, and HPI and increased MTT after the treatment in our study indeed indicated good efficacy. Based on the same reason the increase of HBV, PS, HAP, and HPI and the decrease of MTT in the recurrent patients could be convincingly explained. Poon et al^[34]^ have reported that AFP plays as one of the tumor factors at recurrence for higher level of AFP is observed in patients with recurrence.

Tumor tissues often return lower ADC values than native tissues do, facilitating their detection and characterization.^[13]^ ADC measurement can be quantified through acquiring images with different gradient durations and amplitudes (b-value).^[12]^ Kamel et al^[35]^ reported that tumors demonstrated decreased size and increased ADC values after TACE. Kubota et al^[36]^ demonstrated that an increase in tumor ADC values after TACE correlated with favorable response and tumor necrosis, whereas recurrence or viable tumor presented low ADC values. Changes in HCC after treatment were believed most likely due to the loss of cell membrane integrity and necrosis; specifically speaking, intact cell membranes in viable tumor limit the mobility of water molecules, which was reflected as a low ADC, whereas necrosis enhanced membrane permeability which allowed for increased motion of water molecules and brought about a rise in tumor ADC.^[12]^ The mentioned studies were in accordance with our finding, further validating the value of ADC values on MR-DWI. When performing the MR-DWI, we used 3 b-values to calculate the ADC for accuracy. High proportion of nondiffusional intravoxel incoherent motion (IVIM) was often noticed in images with low b-value.^[37]^ Le Bihan et al^[38]^ also have reported that, with relative low b-values, the signal obtained from large vessels with rapid flow fades away rapidly but small vessels with very slow flow could contribute to the IVIM. Fast BF with rapid wash-out was observed in dynamic gadolinium-enhanced images of HCC lesions; thus, at low b-values, it is supposed to rule out the major part of IVIM contribution.^[37]^ Therefore, the ADC value was regarded valuable only when the b-value was set at 500 or 800 s/mm^2^.

To summarize, this study explored the value of MR-DWI and CT-PI in evaluating efficacy and prognostic value of TACE combined with CT-CT-RFA for the treatment of HCC and concluded that the parameters AFP, ADC value, HBV, PS, HAP, HPI, and MTT of CT-PI had medium value in evaluating the efficacy of the treatment. Further studies may be done on the basis of larger sample size to validate our findings presented here.

## Acknowledgments

The authors thank the Cultivation of High-level Innovation Health Talents of ZheJiang (#2012-241) for the support. The authors also thank the reviewers for their helpful comments on this article.
